# Corrosion inhibition of an aluminum alloy by environmentally derived microbial biofilms

**DOI:** 10.3389/frmbi.2025.1675064

**Published:** 2025-11-25

**Authors:** Zachary T. Burton, Han Liu, Nathan Stumme, Scott K. Shaw, Steven Harris, Simon Laflamme, Kaoru Ikuma

**Affiliations:** 1 Department of Civil, Construction, and Environmental Engineering, Iowa State University, Ames, IA, United States; 2 Interdepartmental Microbiology Program, Iowa State University, Ames, IA, United States; 3 Department of Chemistry, University of Iowa, Iowa City, IA, United States

**Keywords:** corrosion, aluminum, microbial community, biofilm, corrosion inhibition

## Abstract

Microbial biofilms can influence corrosion outcomes on metal surfaces. Though past studies have largely focused on microbiologically induced corrosion, we report here that environmentally derived microbial communities can form biofilms that inhibit the corrosion of an aluminum alloy. Our findings point to the importance of complex microbial communities, which are more likely to be found on metals exposed to natural environments, in determining corrosion outcomes and highlight a potential role of microbial interactions in corrosion inhibition.

## Introduction

Corrosion is a spontaneous electrochemical process causing the deterioration of metals that costs trillions of USD each year across the globe (Koch et al., 2016). Various abiotic environmental factors such as the presence of oxides, hydroxides, sulfides, sulfates, and moisture can cause corrosion (Graedel, 1989). Meanwhile, biotic processes can also cause corrosion; microbiologically influenced corrosion (MIC) that can occur through biofilms formed on metal surfaces is a well-established biological corrosion mechanism (Kip and van Veen, 2015). On the other hand, microorganisms can also inhibit corrosion through a process called microbiologically influenced corrosion inhibition (MICI) (Kalnaowakul et al., 2020). In fact, the definitive role of microorganisms in corrosion remains unclear, with reported outcomes ranging from accelerating corrosion (MIC) (Miller et al., 2018) to inhibiting corrosion (MICI) (Chongdar et al., 2005), or even doing both (Kip and van Veen, 2015).

Though exact processes are yet to be fully uncovered, some potential mechanisms of MICI likely involve microbial respiration, an extracellular polymeric substances (EPS) protective layer, biomineralization, competitive exclusion, and/or the secretion of corrosion inhibitors (Liu et al., 2018; Lou et al., 2021). MICI has been previously observed in simulated marine (Li et al., 2021), soil (Duan et al., 2024), and other (Ruan et al., 2023) environments. However, many MICI studies have focused on biofilms composed of one or two bacterial strains taken from pure culture isolates (Gao et al., 2021; Lou et al 2023; Suma et al., 2019a). Few reports have discussed how fungi play a role in corrosion outcomes (Olivia et al., 2023), and none have investigated how complex environmental microbiomes may impact corrosion outcomes. Indeed, most metal surfaces are exposed to real-world natural settings such as soil and marine/freshwater environments where they encounter a diverse microbial consortia, often in form of biofilms. These biofilms are complex microbial communities composed of multiple bacteria and fungi (Dellagi et al., 2020), where the ecological relevance of such communities lies in the fact that microbial interactions can significantly alter biofilm structure, extracellular polymeric substance (EPS) production, and metabolic activity, thereby shaping corrosion outcomes (Jiao et al., 2021).

Through directly investigating derived microbial communities, this work advances the study of MICI outcomes by bridging the gap between pure culture experiments used in the lab and the complex communities that are encountered in the field which may inform future corrosion management strategies. We report here an initial study aimed at better understanding how complex microbiomes impact the corrosion of an aluminum alloy. This study investigates the MICI outcomes of complex biofilms that were cultured from environmentally derived microbial communities. Biofilm microbial communities derived from local soil and water sources were grown on Al 5005 coupons; the coupons were subsequently exposed to a corrosive aqueous environment and analyzed for corrosion outcomes. Correlations between corrosion outcomes and bacterial and fungal species are reported.

## Methods

### Materials

Flat corrosion coupons made of an aluminum alloy (Al 5005) obtained from Metal Samples Company (Munford, AL) were used in all experiments. The Al coupons were 0.5 inches wide, 3 inches long, and 0.063 inches thick and with a bead blast finish by the manufacturer. The chemical composition of Al 5005 can be found in **Supplementary Table S1**. All chemicals used were ACS grade and obtained from Fisher Scientific (Waltham, MA) unless otherwise reported.

### Acquisition and cultivation of environmentally derived microbial communities

Obtained samples of soils from a landscaped lot and a forest, and surface freshwaters from a creek and a lake were obtained from around Ames, IA. A small amount of each environmental sample (1 g each of soil, 1 mL each of water) was added to 100 mL of 10% LB liquid growth medium as inoculum. The suspended cultures of environmentally derived microbial communities were grown for 72 hours at room temperature (22-24°C) with orbital shaking (160 rpm).

### Biofilm cultivation and corrosion test environment

The Al coupons placed in microscope slide racks were immersed in microaerophilic 10% LB inoculated with each environmentally derived microbial community (1% v/v final inoculation ratio). The coupons were incubated in the presence of the suspended microbial cultures for 72 hours with no mixing to promote biofilm growth. Abiotic control coupons did not undergo this initial inoculation. Following this biofilm growth period, all coupons including the abiotic controls were transferred to a sterile, hypersaline solution (5% NaCl) for 14 days for accelerated corrosion testing. Triplicate coupon specimens were taken for each unique test condition immediately before exposure to the hypersaline salt solution (day 0) and at day 14, and had their biomass sampled before use in profilometry and microscopy analysis to determine corrosion outcomes and microbial community analysis as described below.

### Corrosion analysis using profilometry

The topography measurements of the treated and control Al coupon specimens were performed on days 0 and 14 as well as blanks by using a stylus profilometer. The setup consisted of an extrusion-based frame, and the Cinetics Lynx 3 Motorized Linear Slide that is situated across the ends of the frame. The field of view in the browser-based GUI software was set to approximately 26 mm and the height threshold was set to 1 mm, which prevented the laser from picking up any unnecessary point cloud data at varying heights. The laser-scanned images were saved in the bmg format, in which each pixel represents the height or elevation of a corresponding point on the scanned surface. Surface roughness was characterized by the variation in pixel intensities. Images were loaded into MATLAB and pre-processed by converting them to grayscale to eliminate the effect of brightness variation across the image, making each pixel individually represented by a single intensity value. After that, the height map was derived in double precision to facilitate numerical calculations and prevent potential issues associated with integer arithmetic (Fujiwara, 2019). Root mean square (RMS) roughness and average roughness (Ra), which quantify the average deviation of height values from the mean line of a surface, are widely used metrics for characterizing surface roughness in laser-scanned images (Gharechelou et al., 2018; Suh et al., 2003). A standard deviation filter with a local neighborhood approach was applied in this study to compute the surface roughness to account for RMS and Ra not being statistically robust parameters due to their sensitivity to extreme outliers, and their present limitations in fully capturing anisotropic surfaces (Cech et al., 2013; Webb et al., 2012). Details of the standard deviation filter used here are provided in the SI.

### Corrosion analysis using scanning electron microscopy with energy dispersive X-ray spectroscopy

Al coupon specimens were not coated with metal but only dusted with a can of air to remove loose particles, after which coupons were laid flat onto a plate. The specimens were examined in an FEI Quanta-FEG 250™ SEM at 10 kV. The SEM was operated in low vacuum (or variable pressure) mode with 20 Pa of water vapor. A range of magnifications were used (20x-5000x). Most samples are imaged with backscattered electrons (BSE) for which the brightness of the signal correlates with the density/average atomic number of the material. Microanalysis was done using an Oxford Instruments Aztec™ energy-dispersive spectrometer with an X-Max 80 light-element detector (80 mm^2^ active area). A beam current of ~0.5 nA was used to generate an x-ray count rate of about 15kcps. X-ray maps of 256 × 244 pixels were collected for 10 minutes to show the distribution of the elements.

### Microbial community analysis via amplicon sequencing

Biomass samples were taken from the environmentally derived microbial community inocula, post biofilm growth (day 0), and the day 14 of the corrosion test for DNA extraction. The inocula were collected as cell pellets in triplicate by centrifugation. The biofilms from day 0 and 14 samples were swabbed from the Al coupons using sterile cotton swabs that were dampened in sterile 1X PBS solution. DNA was extracted from the cell pellets and cotton swabs using a DNeasy PowerSoil Pro Kit (Qiagen, Helden, Germany). DNA samples underwent 16S rRNA (V4 region) and fungal internal transcribed spacer (ITS) gene amplicon sequencing on the Illumina MiSeq platform (paired-end 250 bp reads) at the Iowa State University DNA Facility and Metagenom Bio Life Science, respectively (**Supplementary Table S3**). Both DNA libraries were prepped for sequencing using the MiSeq Reagent Kit v2 (Illumina). The resulting amplicon sequencing data were processed using the Mothur SOP (Schloss et al., 2009) for the 16S rRNA gene and the Dada2 pipeline in R (Callahan et al., 2016) for ITS. Both sets of data were then analyzed in R using the following the Mothur MiSeq data SOP (**Supplementary Table S4**).

### Electrochemical analysis

Voltammetry was performed on the benchtop with a CH Instruments CHI660D potentiostat using Al coupon samples as the working electrode, a graphite rod as the counter electrode, and a saturated calomel reference electrode in 0.1 M KCl as the electrolyte solution. LSV (linear sweep voltammetry) was conducted in triplicate on untreated coupons as well as day 0 and 14 Al coupons with their biofilms still intact. LSV was performed from -1 V to 0 V vs. SCE to assess the oxidation onset potential of each Al coupon under varying conditions. Open circuit potentials (OCP) were determined for each coupon sample.

### Statistical analysis

Data were analyzed between test and abiotic control samples using unpaired two-tailed t-tests. Spearman rank correlation analyses were conducted between the differences in surface roughness compared to abiotic controls and the relative abundances of 16S rRNA operational taxonomic units (OTUs) and ITS amplicon sequence variants (ASVs), no multiple-testing corrections (e.g., Bonferroni or FDR) were applied in this analysis.

## Results

### Corrosion outcomes

**Figure 1:**
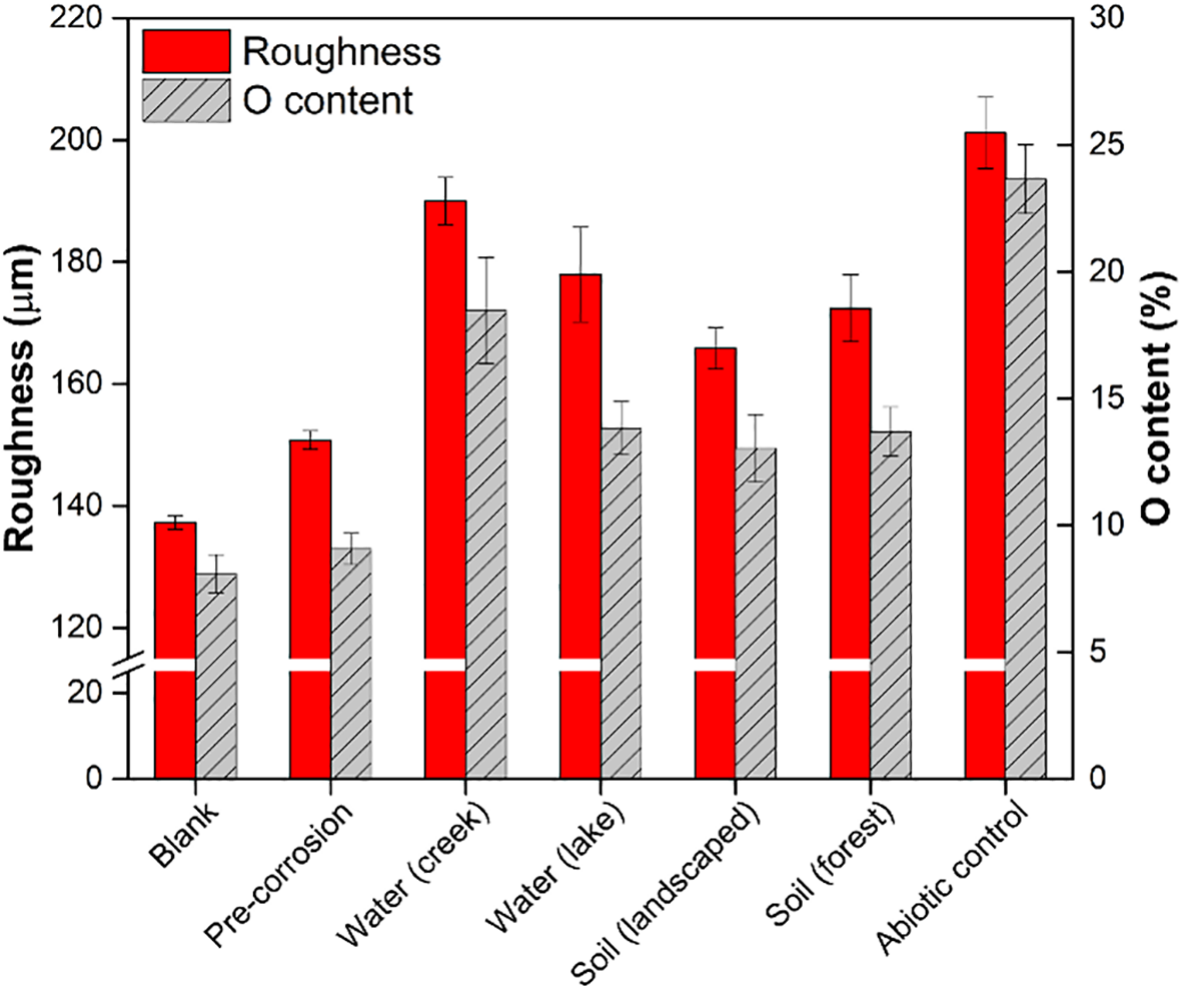
Corrosion outcomes, as a function of surface roughness and O content, of Al 5005 coupons with or without biofilms of environmentally derived microbial communities from different environmental sources following 14 days of exposure to hypersaline solution. Measurements of blank Al 5005 coupons as well as representative coupons with biofilms prior to hypersaline solution exposure (“pre-corrosion”) are also shown. Bars indicate mean values with error bars showing one standard error.

Based on profilometry results, all day 14 coupons with environmentally derived microbial biofilms were observed to be less rough compared to the abiotic control (p<0.05; **Figure 1**). The coupons with biofilms derived from lake and creek water samples were 12 ± 2% (p = 0.075) and 5 ± 8% (p = 0.185) less rough, respectively, compared to the abiotic controls. The coupons with biofilms derived from landscaped and forest soils were 17 ± 7% (p = 0.006) and 14 ± 9% (p = 0.023) less rough, respectively, than abiotic control. As increased metal surface roughness is an indicator of corrosion extents (Toloei et al., 2013), the decreased roughness of the coupons observed here suggests that environmentally derived microbial biofilms inhibited corrosion of the Al alloy surface. It should be noted that pre-corrosion (day 0) coupons with biofilms showed only slightly greater roughness (12 ± 1%) compared to blank Al coupons and significantly lower roughness compared to all post-corrosion coupons (p = 0.009).

SEM-EDS was used to quantify the elemental composition of the Al 5005 coupons (**Supplementary Table S2**). The abiotic control coupons had greater percentages and larger patches of elemental O content compared to the other conditions (**Supplementary Table S2**, **Supplementary Figure S2**), indicating greater extent of corrosion. The elemental O content of the coupons that were exposed to hypersaline solution in the presence of biofilms derived from lake water, creek water, landscaped soil, and forest soil samples were 42 ± 2.1%, 22 ± 4.2%, 45 ± 2.7%, and 42 ± 1.9% smaller than the that of the abiotic control (**Figure 1**). Only the coupons with biofilms derived from soil samples and lake water samples were found to be significantly different from the abiotic control (p < 0.05). However, none of the environmentally derived samples were different compared to each other (p > 0.05).

### Electrochemical analyses

To understand the biofilm’s impact on corrosion resistance, voltammetry was performed to measure the OCP and oxidation onset potential for each Al coupon sample. All coupons showed similar decreases in OCP of 0.03 to 0.05 V over the 14-day exposure period to hypersaline solution (**Supplementary Figure S1**). There was no statistically significant difference between the coupons with and without biofilms, indicating the surface energy for each was similar and changes in surface energy over the 14-day exposure period were minimal.

The oxidation onset potential is an indication of the energy required to oxidize each sample, with a more positive onset potential corresponding to a more stable (oxidation-resistant) surface. In general, the abiotic control coupons showed significant positive shifts in the oxidation onset potential (-0.45 V onset at zero days shifted to -0.20 V onset at 14 days), while the coupons exposed to biofilm showed either flat responses or smaller shifts in oxidation potential (**Supplementary Figure S1B**). This means that the control coupons became increasingly easier to oxidize over time, whereas the biofilm exposed coupons were, on average, less susceptible to these changes. This corroborates the data shown in **Figure 1**, where the roughness and oxygen content are highest in the abiotic control coupons suggesting significant corrosion. Alternatively, the coupons with lower oxygen content and lower roughness values correlate with biofilm exposure. These results, along with the decreases observed in surface roughness and elemental O content of the coupons compared to the abiotic control (**Figure 1**), suggest that the environmentally derived microbial biofilms may have conferred corrosion protection by being more difficult to oxidize.

### Microbial community compositions and their effects on corrosion outcomes

Microbial community analysis was performed on biofilms swabbed from coupons before and 14 days after exposure to hypersaline solution as well as suspended microbial cultures grown from the original environmental samples. Biofilms derived from water samples were dominated by the bacterial class *Gammaproteobacteria* at all 3 time points (**Supplementary Figure S3**). Though the environmental source communities in the soil samples were dominated by *Bacilli* and *Bacteroidia*, the respective biofilms grown on Al coupons were dominated by *Gammaproteobacteria* before and after exposure to hypersaline solution. In these soil-derived biofilms, the presence of *Bacilli* was still observably greater compared to the water-derived biofilms. Meanwhile, the fungal communities had greater shifts in composition over time (**Supplementary Figure S3**).

**Figure 2:**
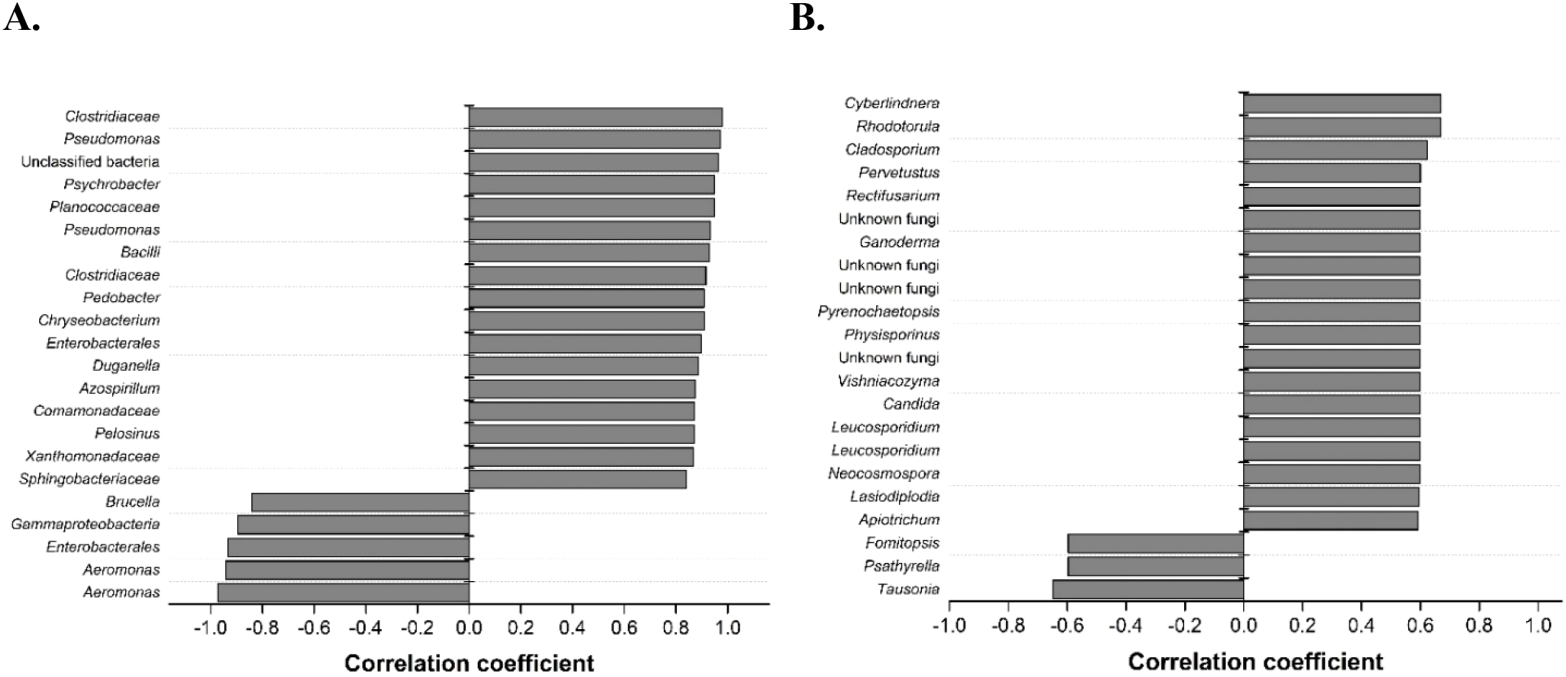
Spearman rank correlation analysis of Al corrosion inhibition with significant bacterial OTUs **(A)** (p < 0.001) and fungal ASVs **(B)** (p < 0.05) after exposure to a hypersaline solution for 14 days. OTUs and ASVs were matched to their respective bacterial and fungal taxa, which were resolved at family and class levels, respectively. More positive correlation coefficients indicate stronger correlation with decreased roughness compared to abiotic controls over the 14-day corrosion period (i.e., corrosion inhibition), whereas more negative correlation coefficients indicate stronger correlation with smaller changes in roughness.

Spearman rank correlation analysis was performed to find possible correlations between specific microbial taxa and changes in surface roughness compared to abiotic controls as a measure of the extent of corrosion inhibition (**Figure 2**). Here, more positive correlation coefficients indicate stronger correlation with decreased roughness compared to abiotic controls over the 14-day corrosion period (i.e., corrosion inhibition), whereas more negative correlation coefficients indicate a stronger correlation with smaller changes in roughness. Biofilm communities at day 14 were observed to have more bacteria and fungi that significantly correlate with decreased roughness (p<0.001 and <0.05, respectively). Positively correlated bacteria include members from the families *Clostridiaceae*, *Bacilli*, *Pseudomonadales*, and *Comamonadaceae*.

**Figure 3:**
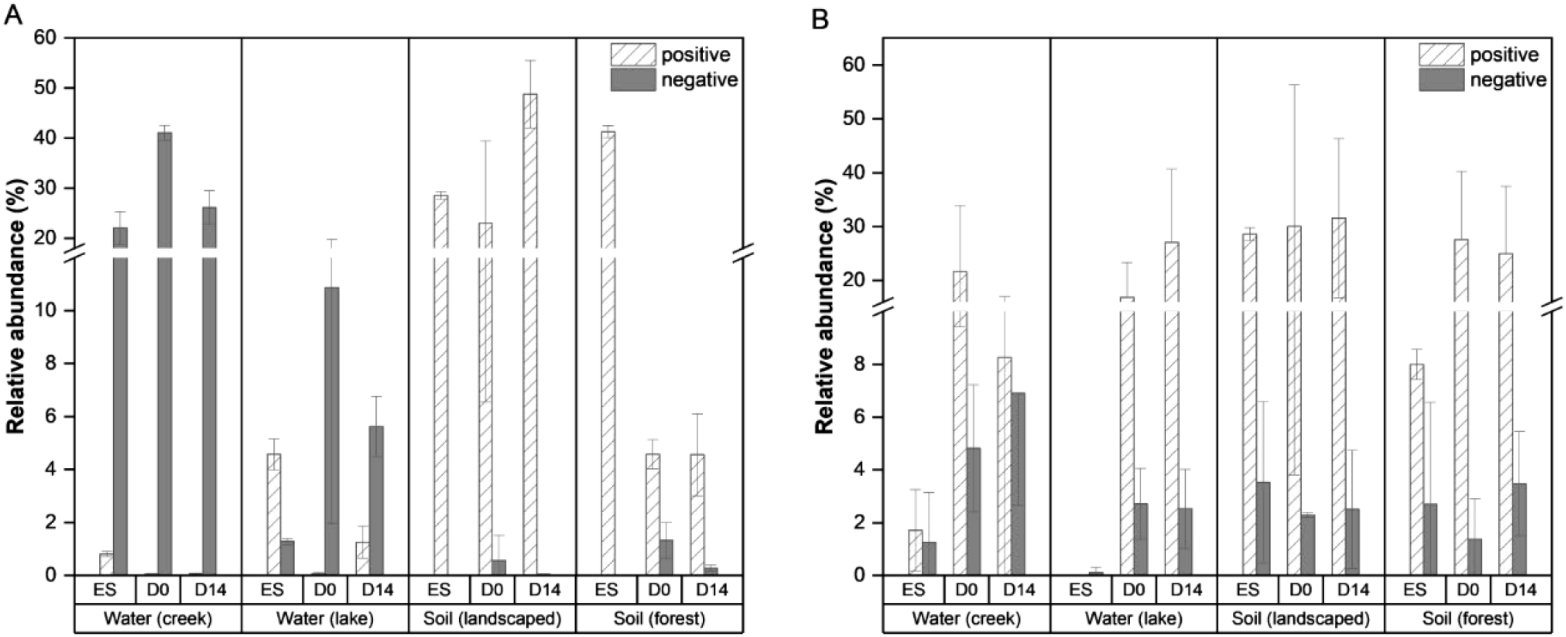
Total relative abundances of bacteria **(A)** and fungi **(B)** with significant positive or negative correlation to Al corrosion inhibition. The total relative abundance is shown as a sum of all OTUs **(A)** or ASVs **(B)** with significantly positive or negative correlation coefficients (**Figure 2**). The environment from which the microbial communities were derived as well as the time points from which samples were taken (ES = environmental source inoculum; D0 = day 0/pre-corrosion; D14 = after 14 days of exposure to hypersaline solution) are shown. Bars indicate mean values with error bars showing one standard deviation.

The relative abundances of the significantly correlated microbial taxa from the environmentally derived biofilms showed different trends for bacteria and fungi (**Figure 3**). Both soil-derived biofilms saw high relative abundance of bacteria that were positively correlated with corrosion inhibition across all 3 time points compared to the water-derived biofilms in which the negatively correlated bacteria had higher abundances (**Figure 3A**). It appeared that in the soil-derived biofilms, these positively correlated bacteria were present in the inoculum at higher levels rather than being strongly selected for during biofilm formation. Positively correlated fungi were in high relative abundance across all 3 time points in all 4 environmental samples with comparatively low relative abundance for the negatively correlated samples (**Figure 3B**). The relative abundances of certain individual strains, specifically *Pseudomonas*, *Pedobacter*, *Bacilli, Sphingobacteriaceae, Cyberlindnera*, *Pervetustus, Neocosmospora, Apiotricum*, *Vishniacozyma*, and *Leocosporidium* increased as the extent of corrosion inhibition increased (**Supplementary Figures S4**, **S5**). These microbes co-occur in the biofilms derived from soils on Al coupons that had increased corrosion inhibition (**Figure 2**). These findings suggest that community composition and microbial interactions play key roles in MICI outcomes.

## Discussion

Microbiologically influenced corrosion inhibition (MICI) has garnered the attention of researchers in engineering, chemical, and microbiological spheres of study. Prior studies have observed that single species pure culture biofilms can have either MIC or MICI outcomes (Suma et al., 2019a). However, biofilms in nature are made up of many bacterial and fungal species that contribute to corrosion outcomes. To elicit a better understanding on how microbiomes affect corrosion, this study investigated the effect of complex environmentally derived biofilms on Al corrosion.

Al alloys are commonly used in marine environments where they are subjected to the corrosive effects of the chloride content and microbial communities present (Ezuber et al., 2008). Measuring the extent of corrosion on Al alloys is typically done using electrochemical methods, here we observed only slight protective shift in oxidation onset potential of the biofilms, though there were no statistically significant differences between the abiotic and biofilm-inoculated coupons. These observations likely reflect the limited sensitivity of OCP to microbial effects in this experimental setup. The oxidation onset potential trends were consistent with the surface analyses; specifically, the abiotic controls exhibited larger positive shifts indicative of greater oxide layer formation, whereas biofilm-exposed coupons showed comparatively smaller changes. However increased metal surface roughness and O content, obtained from profilometry and SEM-EDS respectively, can also be used as an indicator of corrosion extent (Barranco et al., 2010; Toloei et al., 2013; Zhang et al., 2022, 2023). Here decreased roughness of the coupons observed on all inoculated coupons, along with lower O content observed as well in the soil and lake water samples suggest that environmentally derived microbial biofilms exhibit corrosion inhibition capabilities for the Al alloy surface. Nevertheless, we acknowledge that a larger scale study coupled with more comprehensive electrochemical experiments such as linear polarization resistance (LPR) and EIS would provide additional confirmation, and we note this as a direction for future work.

Though most MICI studies thus far have focused on single or dual species biofilms, this present study highlights how bacteria and fungi do not exist in isolation of each other on metal surfaces exposed to most environmentally derived microbiomes, and how they interact with each other may be important in determining MICI outcomes and how the biofilm functions (Khan et al., 2020; Li et al., 2021). These factors can also affect how the microbiome present in the biofilms can influence corrosion outcomes. In this study the fungal communities saw large shifts in composition over time, which falls in line with previous observations of fungal communities that were exposed to hypersaline conditions (Yang et al., 2023). In pure strain studies, the introduction of different bacteria can make a significant impact on corrosion outcomes (Suma et al., 2019a). Here bacteria of the class *Bacilli* were also observed to be in greater abundance in the MICI capable soil inoculated samples, and members of *Pseudomonas* also saw increases in relative abundance as the degree of corrosion inhibition increased (**Supplementary Figure S4A**). Previously, members of classes *Bacilli* and *Pseudomonas* had been reported to protect against corrosion, possibly through the production of EPS and other corrosion inhibitors (Li et al., 2019; Liu et al., 2023; Örnek et al., 2002; Suma et al., 2019b; Zou, 2007), supporting our observation that the soil-derived biofilms had greater corrosion protection potential.

Additionally, among the observed fungal taxa, *Cyberlindnera*, *Neocosmospora*, and *Vishniacozyma* have previously been implicated in biomineralization or biofilm structural stabilization processes that may reduce metal dissolution under saline stress (Yang et al., 2023). The consistent presence of positively correlated fungal taxa across all environmental samples suggests that fungi may play a more general role in corrosion inhibition than bacteria, whose effects appeared stronger in soil-derived communities. The observations of possible EPS production and biomineralization by the environmentally derived biofilms suggest that both bacterial and fungal community members may contribute to biofilm stabilization and MICI outcomes. Furthermore, with the primary focus of this study being to investigate environmentally derived complex communities as an exploratory step toward ecological relevance, it has the limitation of direct comparison to single- or dual-species biofilms. Future studies should incorporate side-by-side evaluations of pure culture and mixed-community biofilms to better delineate the unique functional contributions of complex environmental microbiomes to MICI. These studies may look to expand upon these findings with metagenomic sequencing to gain a deeper understanding of the genetic mechanisms that may be at play within these systems. Additionally, future studies employing metagenomic, metabolomic, and co-culture approaches are needed to obtain a better understanding of how fungi contribute to corrosion inhibition.

Our findings suggest that microbial interactions likely play an important role in shaping MICI outcomes, but the mechanistic basis remains to be fully elucidated. Previous studies have observed that bacterial-fungal interactions in microbial communities can influence nutrient cycling and overall microbial diversity (Jiao et al., 2021). These competitive interactions could lead to niches within the microbiome to exclude MIC organisms (Lai et al., 2020) and shifting the balance toward corrosion inhibition. Additionally, the utilization of biomineralization and the production of EPS and its composition may also be affected through microbial interactions (Hwang et al., 2017), which in turn could affect an environmentally derived biofilm’s MICI capabilities. The effect of microbial interactions and composition was observed in how the relative abundance of fungal species positively correlated with corrosion inhibition was high in all biofilms, while positively correlated bacterial species were high only in the soil-derived biofilms. All four environmentally derived biofilms had some corrosion inhibiting effect suggesting the corrosion inhibiting fungi may be having some effect in the bacterial-fungal interactions to allow for any MICI outcome. Meanwhile in the soil derived biofilms where both of the positively correlated with corrosion inhibition bacteria and fungi are in high abundance, we observed a stronger MICI effect. Although our amplicon sequencing results identify taxa correlated with MICI, future studies will need to disentangle these microbial interactions directly, for example by employing synthetic microbial consortia, metagenomic sequencing, or metabolomic analyses, to determine the precise pathways by which microbial interactions influence corrosion outcomes.

Though a limited number of environmental samples were tested, this exploratory study looked into samples representative of both water and soil environments to provide an initial view of possible microbial corrosion outcomes of Al alloys exposed to naturally occurring microbiome, and encourages further larger scale research into this area as MICI may be a relatively common occurrence in the natural environment. Another limitation of the present study is the relatively short exposure period of 14 days under a single set of environmental conditions (room temperature, microaerophilic growth, and hypersaline exposure). While hypersaline conditions were used to accelerate corrosion processes, longer timescales are often necessary to observe more severe corrosion outcomes and for observation of competition between the biofilms and traditional MIC organisms such as sulfate-reducing bacteria. Such longer timescale studies should incorporate “real-world” settings such as fluctuating temperature, pH, and salinity, all of which can influence biofilm composition and corrosion outcomes. Therefore, the observed inhibitory effects represent an initial indication of MICI potential, but longer-term studies incorporating extended incubation periods and direct competition assays will be needed to confirm the durability and ecological relevance of these outcomes. A further limitation of the present study is the relatively small sample size, as most analyses were conducted on triplicate coupons across all experimental conditions. However, the small number of replicates reduces statistical power and may limit the robustness of some conclusions. Future studies should therefore incorporate larger sample sizes and additional replicates to strengthen statistical confidence and better capture variability in microbial community effects.

Overall, this study found that environmentally derived complex biofilms contained various microorganisms that were either strongly positively or negatively correlated with corrosion inhibition, suggesting that these outcomes are partially controlled by microbial interactions. The co-occurrence of specific microbial strains such as *Pseudomonas* sp. and *Cyberlindnera* sp. was strongly correlated with increased corrosion inhibition, pointing to the potential role of microbial interactions in MICI and biological corrosion outcomes. Though only four environmental sources were tested here, this study provides an initial demonstration that complex, naturally derived microbiomes can influence corrosion outcomes of Al alloys. Future investigations incorporating a broader diversity of environments will be essential to determine the consistency and mechanisms of MICI across ecological contexts.

## Data Availability

Sequencing data for this article can be found under the NCBI BioProject PRJNA1153592, which includes all 16S and ITS sequences. Other data, including information on how surface roughness was computed, packages required for R, and the pipelines for both Mother and Dada2, can be found in **Supplementary Information**.
